# Transpiration Dynamics of Esparto Grass (*Macrochloa tenacissima* (L.) Kunth) in a Semi-Arid Mediterranean Climate: Unraveling the Impacts of Pine Competition

**DOI:** 10.3390/plants13050661

**Published:** 2024-02-27

**Authors:** Iván Pérez-Anta, Eva Rubio, Francisco Ramón López-Serrano, Diego Garcés, Manuela Andrés-Abellán, Marta Picazo, Wafa Chebbi, Rocío Arquero, Francisco Antonio García-Morote

**Affiliations:** 1 Environment and Forest Resources Group, Renewable Energy Research Institute, University of Castilla-La Mancha, Campus Universitario s/n, 02071 Albacete, Spain; ivan.perez@uclm.es (I.P.-A.); fco.lopez@uclm.es (F.R.L.-S.); diego.garces@uclm.es (D.G.); manuela.andres@uclm.es (M.A.-A.); martaisabel.picazo@uclm.es (M.P.); wafa.chebbi@uclm.es (W.C.); rocio.arquero@uclm.es (R.A.); 2 Applied Physics Department, University of Castilla-La Mancha, Campus Universitario s/n, 02071 Albacete, Spain; evamaria.rubio@uclm.es; 3 Higher Technical School of Agricultural and Forest Engineering, University of Castilla-La Mancha, Campus Universitario s/n, 02071 Albacete, Spain

**Keywords:** alpha grass, water exchange, Aleppo pine, plan competition, semi-arid steppes, leaf senescence

## Abstract

*Macrochloa tenacissima* (*M. tenacissima*), or esparto, is a perennial tussock grass that coexists with *Pinus halepensis* (*P. halepensis*) in semi-arid Mediterranean woodlands. This research was carried out to explore diurnal transpiration at leaf level in esparto grass under different levels of pine–esparto competition and in contrasting environmental soil water conditions. The measurement period spanned from the summer of 2020 to the spring of 2021. The relationship between transpiration and competition was conducted in open and closed *P. halepensis* stands, and the type of leaf (green, senescent) and the maturity of the esparto grass were taken into account. We observed a higher control of transpiration in green leaves, and the correlations between the transpiration and pine competition were noted exclusively in this type of leaf. Our results demonstrated a significant impact of pine competitors (closed stands) on the transpiration of esparto grass, particularly during seasons characterized by scenarios of high water demand: the summer drought period and the commencement of the growing and flowering period (spring). Furthermore, our findings revealed a greater response to transpiration in mature bushes compared to young ones under severe water stress, indicating a higher adaptation to drought by esparto as it ages. Although our results confirmed that PAR increased transpiration in all seasons and in both stands, which is attributable to the heliophilia of esparto grass, the site effects on transpiration could also be attributable to competition for water, especially during periods of drought. These results may have important implications for the dynamics and management of these semi-arid mixed woodlands, as well as the planning of reforestation programs aimed at restoring esparto grass formations.

## 1. Introduction

Water is the principal factor limiting vegetation growth in semi-arid climates; consequently, drought constitutes the most severe environmental stress for plants in these ecosystems [1]. Understanding how species have adapted to water availability is crucial for forest ecosystem management [2], especially in areas with limited water resources, such as Mediterranean semi-arid climates, where low soil water content during the dry periods is the main factor that limits plant growth [3].

Among the adaptations that allow survival under water deficit conditions, the control of transpiration is the best-known physiological plant response [4]. Transpiration is the process through which water moves from the soil, passes through the plant, and is released into the atmosphere as water vapor from the leaves [5,6]. The two main physiological effects of transpiration are cooling and the provision of water to leaves for photosynthesis, i.e., vegetal growth [7].

The response of plant transpiration to environmental conditions is extremely complex and is dependent on multiple factors. For example, leaf temperature, photosynthetically active radiation (PAR), relative humidity, and air velocity affect water exchange from leaves, illustrating the intricate nature of this process [8]. At the stand level, transpiration varies across species, meteorology, and soil conditions [7,9,10,11]. The link established between water availability and soil parameters has consequently made soil water status an important factor in the study of transpiration dynamics, particularly in scenarios of soil drying [12]. Furthermore, studies based on leaf parameters have often proven to be inadequate when explaining the transpiration responses in Mediterranean sites; these responses are characterized by strong seasonality [3], particularly during the summer drought [13], because variation in transpiration depends on seasonal soil water conditions [10]. In arid environments, water use efficiency during the growing seasons is primarily controlled by transpiration [14].

Seasonal rainfall limits physiological processes and plays a pivotal role in the augmentation of soil water content and the increase in transpiration [9,15]. Clear evidence now suggests a metabolically mediated response (“hydroactive feedback”) between stomatal responses and soil drought, possibly involving abscisic acid production in leaves [12]. This implies that in arid environments, small rainfall pulses (0–5 mm) would lead to increased plant transpiration [15]. Given all this evidence, there has recently been an increase in studies focusing on the relationship between soil water content and transpiration [2,9,14]. Ungar et al. [16] cited the soil water content as the primary driver of transpiration.

However, the effect of soil moisture conditions on plant transpiration in the Mediterranean basin has often been neglected, and relatively little information is available on typical Mediterranean species regarding this issue. This is exemplified by *Stipa tenacissima* L. (“esparto”; Poaceae), a perennial tussock grass widely distributed in the semi-arid ecosystems of the southwestern Mediterranean basin [17]. Esparto grass is a robust, large herbaceous species, with a wide tussock ranging from 45 to 210 cm [18]. The leaves of esparto grass are thin, ribbon-like, smooth, and shiny [19], forming a specialized leaf tissue that facilitates leaf rolling. This structure plays a crucial role in the control of the water balance of leaves under drought conditions [20].

Another noteworthy characteristic of this bush is the presence of senescent leaves, which also contribute to its survival and the completion of the plant life cycle under drought stress [21]. The tussock exhibits different leaf age classes, including green, senescent, and dead leaves [18]. Ramírez et al. [22] demonstrated the necessity of considering leaf senescence when studying transpiration in *M. tenacissima*. The importance of foliar self-shading as a structural photo-protective mechanism in this species has also been observed [23,24], and Ramírez et al. [24] noted diverse physiological responses to water conditions in *M*. *tenacissima* individuals based on bush size.

*M. tenacissima* frequently coexists with *Pinus halepensis* Mill. (*P. halepensis*) in semi-arid areas of southeastern Spain, where the pine has been the most widely used tree in reforestation [17]. In these Mediterranean forests, *M. tenacissima* is the dominant species present in the herbaceous layer of *P. halepensis* stands. However, important differences in pine density and esparto cover can be observed in the same area, as the result of the regressive succession from an initial closed forest dominated by *P. halepensis* [17]. In southeastern Spain, the forest authorities encouraged the elimination of *P. halepensis* to favor the expansion of esparto grass during the second half of the last century, due to the high industrial demand for esparto grass fibers. As this economic activity declined (by the 1970s, during the last century), the progressive succession to the original pine forests became difficult due to soil erosion, severe droughts, and grazing pressure [25].

Consequently, the resulting woodlands are structured as small closed stands that have recovered in that they have better quality soil with little slope; they exist alongside open stands dominated by esparto grass, where only dispersed pine trees are growing. These mixed formations reflect important plant–environment interactions (as competition) that permit the evaluation of the adaptabilities of Stipa to succession and environmental changes [26].

Another relevant aspect of the investigation of this species is its susceptibility to climate change. The Mediterranean region, which is identified as a hotspot that is vulnerable to increased droughts [3], is expected to face adverse effects on the survival and growth of esparto bushes under the predicted scenarios [27]. In this context, Krichen et al. [28] observed a negative impact on the plant’s total biomass and leaves as rainfall became scarcer. Smaller esparto bushes may be particularly sensitive to drought; this could lead to a reduction in the genetic diversity of the species [24]. Pugnaire et al. [29] and Ramírez et al. [22] observed that *M. tenacissima* halts leaf extension following a decrease in water content. Ghiloufi et al. [30] identified a negative relationship between aridity and the cover of vegetation dominated by esparto. These findings suggest that an increase in drought could contribute to the loss of esparto cover in the Mediterranean basin.

In semi-arid regions, the arrangement of bush species into “patchy vegetation”, which is characteristic of steppes dominated by esparto grass, represents an additional adaptation related to the optimization of soil water [1,18,31]. However, in forest ecosystems dominated by pine trees with an understory of bush species, such as esparto grass, the adaptation of plants to environmental conditions is also influenced by intrinsic factors in the forest structure, such as density, competition, maturity, and size [32]. As a result, transpiration in these mixed pine bush forests depends not only on environmental conditions but also on canopy density and structural features [10].

In this scenario, both facilitation and competition between esparto grass and *P. halepensis* influence plant physiological responses [17]. However, competition becomes a crucial factor, influencing water availability, size, growth, and foliage exposure to incident radiation or PAR [10]. The competition for resources within different strata of a semi-arid ecosystem affects grass transpiration, which involves water absorption by the tree stratum and indirectly impacts the bush through reduced irradiance caused by shadows cast by trees [17,29]. The study of ecophysiological variables at the stand level also requires consideration of plant structure, as plant water exchange varies with plant size, leaf surface area, amount of senescent leaves, and distance from competitors [33,34].

Therefore, in planning our experimental layout and considering the importance of plant competition in physiological processes, such as the transpiration rate, we hypothesized that interspecific pine competition for water resources (characterized by soil humidity) and light can affect water fluxes in esparto grass. We also predict that the type of leaf (green or senescent) and the maturity of esparto grass could influence the transpiration rate responses. The inherent high seasonality in the Mediterranean climate compels us to test these hypotheses throughout the four seasons of the year.

Consequently, the aims of this study are twofold: (1) to investigate the seasonal variation in the transpiration rates in *M. tenacissima* in relation to leaf type (green or senescent), the stand inter-specific competition (open sites without *P. halepensis* competition and closed sites with pine competence), and the maturity of esparto bushes (young or mature); (2) to determine the effects of soil water on the daily transpiration of *M. tenacissima*, along with the interactions occurring due to leaf type, site or competence, and the maturity of bushes throughout the four growing seasons under a Mediterranean semi-arid climate. The findings from our study have the potential to provide accurate predictions regarding the responses of *M. tenacissima* to shifts in climate or competition conditions.

## 2. Results and Discussion

### 2.1. Seasonal Trends in Microclimatic Soil Variables and Transpiration Rates

**Figure 1:**
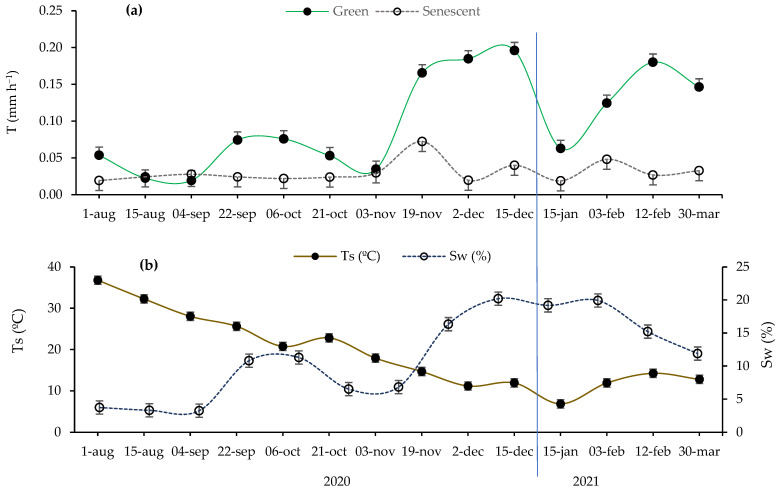
(**a**) Variation in measured transpiration rate (T; mm h^−1^) in both green and senescent leaves throughout the study period’s seasons and (**b**) variation in soil microclimatic variables during the sampling period, including soil water content (Sw; %) and soil temperature (Ts; °C). Error bars: standard error. The vertical blue line separates the sampling days of the years 2021 and 2022.

Broadly, Figure 1 illustrates that our research was carried out in four seasons characterized by varying transpiration (T; mm h^−1^), soil water content (Sw; %), and soil temperature (Ts; °C), according to the seasonality of Mediterranean climates. The microclimatic seasonality (Sw, Ts) causes transpiration to exhibit significant oscillations throughout the seasons. However, this phenomenon is only clearly evident in green leaves (Figure 1a). In all the seasons, except for summer, the transpiration rate was clearly higher in the green leaves compared to the senescent leaves, which maintained their daily transpiration below 0.1 mm h^−1^ during all the measurements.

As green leaves are the physiologically active leaves, the T of these leaves is correlated with the biological cycle of esparto grass, which is characterized by two growing seasons (fall and spring) and two latent seasons (winter and summer) [14,27]. During the summer of 2020, the soil moisture frequently remained below 5%, creating conditions conducive to water stress (Figure 1b). As a consequence, the T of the green leaves remained below 0.1 mm h^−1^, with no evident differences in transpiration that depended on the type of leaf (Figure 1a). When soil water decreases, the stomata may open only partially, and transpiration may occur only through the cuticle or lenticels [10]. This process can lead to the complete suppression of CO_2_ assimilation [35].

In autumn, the T of the green leaves exhibited an increase following soil water recovery (Figure 1a,b). This trend continued until the climate became cold enough to hinder plant growth (winter), resulting in another decrease. Afterward, there was an increase in T coinciding with the opening of the buds and the expansion of the leaves (spring 2021). However, by the end of winter, we observed significant water exchange activity. In the study area, winters are characterized by relatively elevated temperatures, and esparto grass may exhibit acclimation to local environmental conditions, such as increased transpiration.

### 2.2. Seasonal Variation in Microclimatic Soil Variables and Environmental Conditions in Function of Site

**Figure 2:**
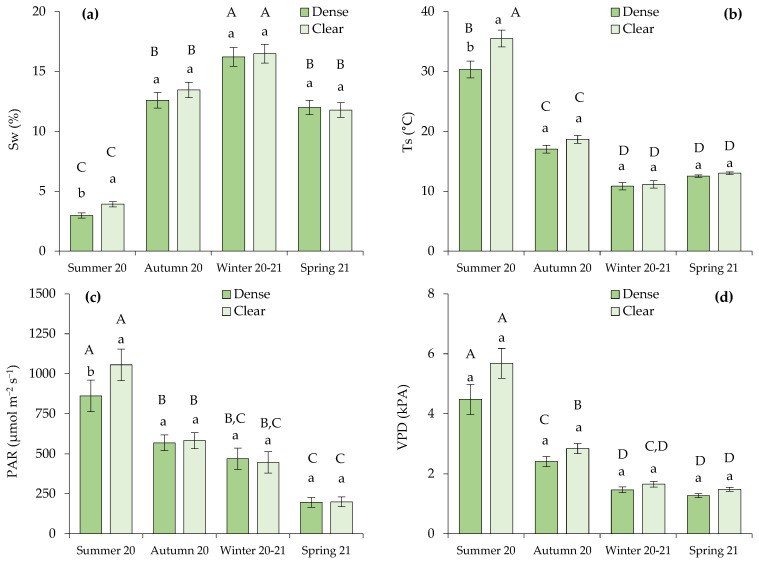
Microclimatic soil variables measured in the sampling bushes, defined by (**a**) soil water content (Sw; %) and (**b**) soil temperature (Ts; °C), along with environmental conditions correlated with the transpiration rate, specifically (**c**) photosynthetic active radiation (PAR; µmol m^−2^ s^−1^) and (**d**) vapor pressure deficit (VPD; kPa). Distinct lowercase letters indicate statistically significant differences within one season, while different uppercase letters signify statistically significant differences (*p* ≤ 0.05; LSD test) between seasons. Error bars represent standard errors.

Differences between open and closed stands in the analyzed parameters were only observed in the measurements of Sw, Ts, and photosynthetically active radiation (PAR) that were registered in the summer (Figure 2a–c). During this dry season, the cited parameters were significantly higher in the clear site.

We also observed that the most contrasting conditions for all the analyzed variables occurred during the summer of 2020 compared to the rest of the seasons (Figure 2). Lower Sw and higher Ts, PAR, and vapor pressure deficit (VPD) were measured during drought conditions. The VPD varied significantly depending on the season, but not on the site within each season, as VPD represents a forest-scale environmental variable.

### 2.3. Correlations between Pine Competition, Soil Microclimatic Variables, Environmental Conditions, and Transpiration Rate of Green Leaves

It is noteworthy that the competition index (CI) had a significant and negative impact (*p* < 0.05) on Sw and T during the summer and spring seasons (Table 1), which represent the dry and most important growing seasons, respectively. This indicates that the closer the pines are to the bushes, the lower the water content of the soil and the water exchange (T) from the esparto bush. Additionally, the CI negatively affected the Ts and the PAR in summer, although the correlation was not significant for the incident radiation (95% level).

The highest number of correlations (Table 1) was noted under the conditions of restricted soil water availability (summer). The T of the green leaves exhibited positive and significant correlations with Sw and PAR during this dry season This confirms that *M. tenacissima* can respond opportunistically to pulses of rainfall within the drought season, as shown by Pugnaire et al. [29]. VPD appears to exhibit a similar behavior to PAR in summer.

In Table 1, it is also observed that the T of the green leaves exhibited a positive and significant relationship with Sw throughout all the seasons, except during winter (dormancy season). Conversely, the Sw and Ts trends show a consistent, negative relationship: as soil temperature increases, soil moisture decreases. Therefore, a single soil microclimatic variable should be adequate for studying the effects of edaphic parameters on T, and the soil Sw appears to be more suitable due to the faster, positive response of transpiration to changes in soil humidity, including during drought periods. Soil water is considered a variable that is expected to exhibit a strong relationship with T and water availability [16].

In addition, we observed a consistent positive correlation between PAR and T across all the seasons, with the strongest relationship (highest correlation coefficient) observed in spring. These results underscore the reliance of transpiration in *M. tenacissima* L. (heliophilic species) on solar radiation, particularly in soils characterized by high water content [22].

However, under severe drought conditions (such as those in summer), the stomatal opening can be more responsive to intracellular CO_2_ and abscisic acid concentrations in the leaf than to environmental factors such as PAR [36,37]. Additionally, previous studies of various *Macrochloa* species [38,39] have demonstrated high levels of water loss from the cuticle during episodes of water stress, despite the accumulation of waxes in the cuticle, which aids in the prevention of water exchange through non-stomatal transpiration [40]. Hence, transpiration in summer could be attributable, in part, to water loss through the leaf cuticle, which also depends on soil water [41].

### 2.4. Biometric Characteristics of Sampling Bushes: Plant Structure

**Figure 3:**
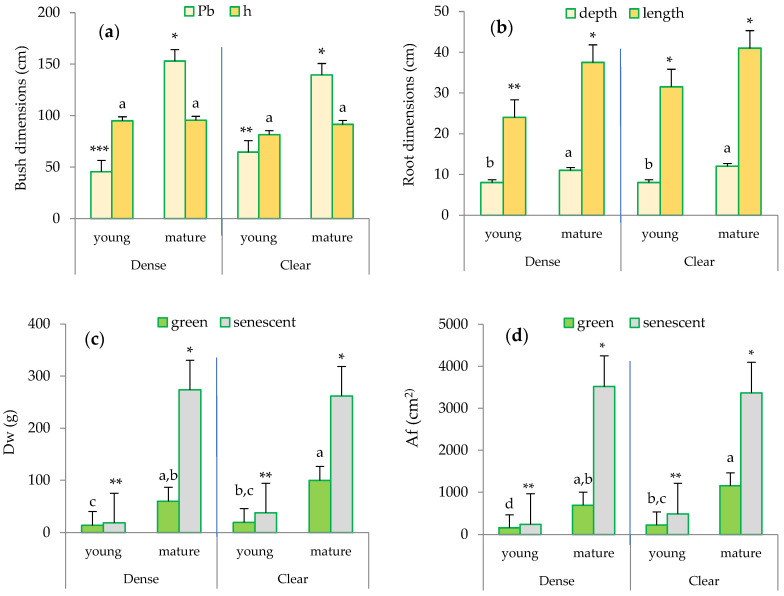
Biometric characterization of the esparto grasses sampled in the study area based on leaf type, site, and maturity: (**a**) bush dimensions: Pb (perimeter at the base in cm), h (height of the bush in cm); (**b**) root dimensions (cm): depth and length; (**c**) Dw (foliar dry weight in g); and (**d**) Af (foliar area in cm^2^). Distinct lowercase letters indicate statistically significant differences (*p* ≤ 0.05; LSD test) between bushes in terms of height (**a**), root depth (**b**), foliar biomass for green leaves (**c**), and foliar area for green leaves (**d**). Different numbers of asterisks signify statistically significant differences (*p* ≤ 0.05; LSD test) between perimeter at the base (**a**), root length (**b**), foliar biomass of senescent leaves (**c**), and foliar area for senescent leaves (**d**). Error bars represent standard errors.

While the height (h) of the clumps selected by size class was similar in both sites, the maximum difference in the perimeter at the base (Pb) of the bush was observed between the mature esparto grass within the clear stand and the young individuals in the dense site (Figure 3a).

Regarding the root system (Figure 3b), significant differences were observed between the mature and young esparto grasses in both sites. Notably, the root length was higher in the mature esparto grasses measured in the clear sites. Furthermore, the senescent leaves exhibited higher biomass (Dw; g) and leaf area (Af; cm^2^) compared to the green leaves (Figure 3c,d), and a significant finding with regard to the foliar structure in the green leaves was the considerably higher Dw and the associated Af in the mature bushes compared to the young esparto grass in both sites. Additionally, the most noticeable difference was observed in the Af between the mature esparto grass growing in the clear sites and the young bushes within the closed stands.

These findings related to root and foliar structures lead us to infer that the transpiration in the two types of bushes differs due to the differences found in the plant organs integral to the transpiration process; this is because they reflect the combination of adaptive strategies to soil water uptake (root development) and light interception (leaf area) [4,26]. The biometric changes observed in *M. tenacissima* as it ages are also indicative of strategies that enhance water use efficiency; these strategies are typically found in mature, more drought-adapted plants and potentially increase transpiration [42]. For example, the accumulation of senescent biomass contributes to the enhancement of the micrometeorological conditions within the plant [24,34,43]. These strategies also underscore the significance of quantifying the contribution of senescent leaves to transpiration and to scale transpiration at the bush or ecosystem levels [24].

### 2.5. Effects of Leaf Senescence on Transpiration Rate

Table 2 presents the key statistics (*p*-value) for the selected linear mixed model with AIC= 190.43 and BIC = 225.21; this model considers the T of both the green and the senescent leaves as the dependent variable. The most notable result was that leaf type significantly affected T (*p* < 0.001). The average T of the green leaves (0.102 ± 0.005 mm h^−1^) was significantly higher (*p* < 0.001) than that of the senescent leaves (0.031 ± 0.001 mm h^−1^) (mean ± standard error). These results are consistent with the findings reported by Haase et al. [27], who observed that the transpiration rates for senescent leaves were lower than those obtained for green leaves.

**Figure 4:**
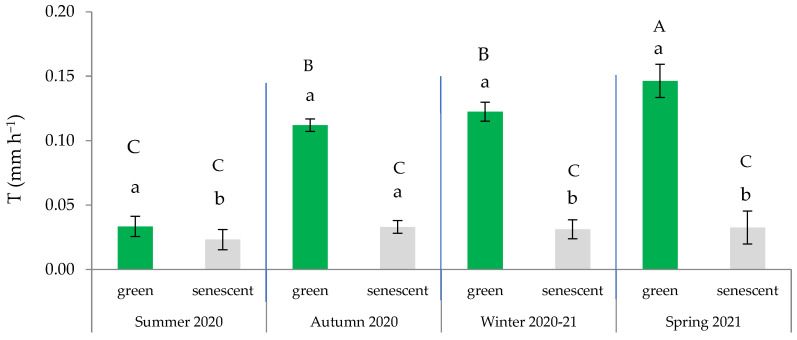
Variation in transpiration rate across the four seasons based on the type of leaf (green or senescent). In each season, distinct lowercase letters indicate statistically significant differences (*p* ≤ 0.05; LSD test), while different uppercase letters signify statistically significant differences (*p* ≤ 0.05; LSD test) between seasons. Error bars represent standard errors.

Additionally, the leaf × season interaction was significant (Table 2), indicating that the measured transpiration significantly differed based on the type of leaf within spring, autumn, and winter. These findings confirm a strong seasonality in the water regulation that is dependent on the type of leaf. Consequently, the higher T was observed in spring and was primarily attributable to the activity of the green leaves (Figure 4). On average, the T of the green leaves during the summer remained below 0.050 mm h^−1^, which was comparable to that of the senescent leaves in all seasons. These findings align with those obtained by Ramírez et al. [42], indicating a latent activity period in summer, except for during punctual pulses of rainfall, which can activate transpiration.

The significant activity of water exchange during the winter (local acclimation) suggests stomatal control of transpiration by green leaves throughout the four seasons, with a notable emphasis during the summer to prevent water loss through the stomata [6]. However, in summer, the loss of water from senescent leaves could also be crucial as a cooling mechanism to counteract the high levels of solar radiation [29,33,44]. The transpiration that is independent of stomatal regulation could be attributable to water loss through the foliar cuticle [38,42] and/or the adsorption and subsequent evaporation of dew water by the cellulose fibers [45].

### 2.6. Effects of Site and Maturity on Transpiration in Green Leaves

Table 3 shows that the season also significantly affected T in the green leaves (*p* < 0.001), and the sampling time defined significant effects within seasons; this was particularly notable for the site in spring and summer, and the site × maturity interaction in summer.

**Figure 5:**
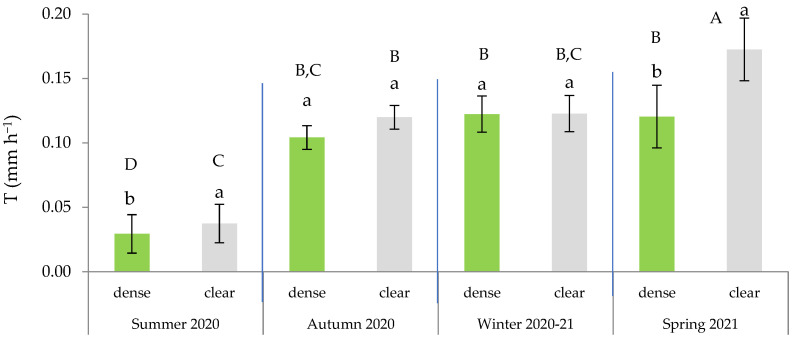
Transpiration rate (mm h^−1^) in green leaves across the four seasons and in the function of the site. In each season, distinct lowercase letters indicate statistically significant differences (*p* ≤ 0.05; LSD test), while different uppercase letters signify statistically significant differences between seasons (*p* ≤ 0.05; LSD test). Error bars represent standard errors.

According to the ANOVA results, the highest T was recorded in the bushes farthest away from pine competitors (clear site) at the beginning of the growing season, during spring (Figure 5). We also observed that during the drought period, the T in the open site was significantly higher than in the dense stands, where there was competition between the pines and the esparto grass.

**Figure 6:**
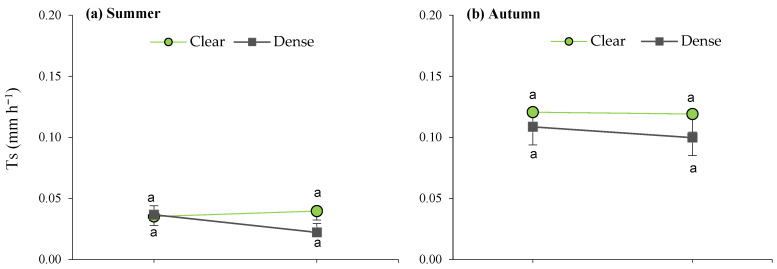

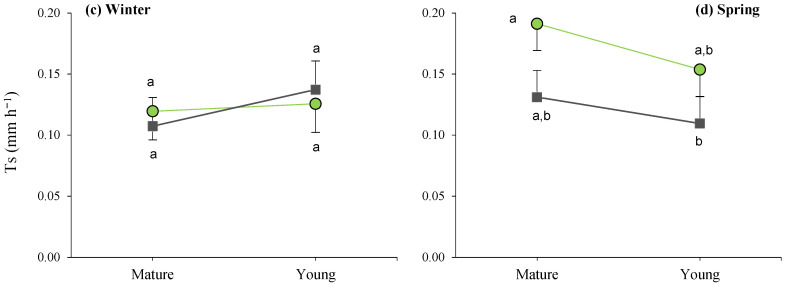
Interaction plots between site and maturity across the four seasons for the transpiration rate in green leaves. Means sharing the same letter within each season are not significantly different at a 95% probability level (LSD test; *p* ≤ 0.05).

When analyzing the site × maturity interactions across the seasons (Figure 6), it can be observed that the lowest average daily transpiration was recorded in the young plants, as well as during the summer in the dense stand, while the remaining average T values were similar within seasons, irrespective of maturity.

Furthermore, during spring, the site effects led to significant differences between mature bushes in the clear stands when compared to young esparto grass in the closed sites (Figure 6). In a manner that is consistent with the aforementioned observations, these findings indicate that transpiration in *M. tenacissima* decreased in younger individuals and in situations of competition with pines, particularly during periods of high water demand. Similar results were observed by Ramírez et al. [24] during the water-stress season; they detected significant differences in transpiration among tussock-size classes in stands with low *M. tenacissima* cover. An explanation for this is that, with age, esparto grass gradually acclimatizes to stressful environments to optimize resource utilization, such as water and light [33].

In this context, although self-shading could be greater in large bushes compared to small bushes [33], water use efficiency is higher and photoinhibition is lower in large esparto tussock [24]. Additionally, since our results were obtained from leaves totally exposed to light at the top of the bush, the self-shading effect was attenuated, thus emphasizing the effects of other factors, such as pine competition.

Finally, as root depth was higher in the mature bushes (Figure 3b), we hypothesize that the higher transpiration measured in mature esparto could, in part, be attributable to a more developed root system. This is consistent with the findings of Ramirez et al. (2008) [24], who observed that small tussocks in the early development stage were more vulnerable to water stress. Considering the significant influence of bush size on run-off retention [25], mature individuals could also maintain greater water reserves in the soil.

### 2.7. Seasonal Effects of Soil Water and Competition on Transpiration in Green and Senescent Leaves

Table 4 presents the significant parameters defining the combined exponential power model (Model 1). As mentioned in the methodology section, the model incorporates site as a confounding effect of pine competence on both soil water and incident radiation.

The resulting models showed that T was independent of the site (pine competition) and Sw for the senescent leaves (Table 4). This represents a constant value of T (e^β0^) for each season, suggesting minimal physiological activity. Conversely, Sw significantly influenced the T in the green leaves throughout all the seasons, impacting the overall yearly model. As is evident in the coefficients presented in Table 4, T increased with Sw, except during winter, which is the dormancy season.

Table 5 presents the models fitted for each site, categorized by leaf type, and it summarizes the microclimatic conditions during the study period. Notably, the Sw seems to play a crucial role in governing T, especially during warm temperate periods like spring and autumn, as well as during the drought season.

**Figure 7:**
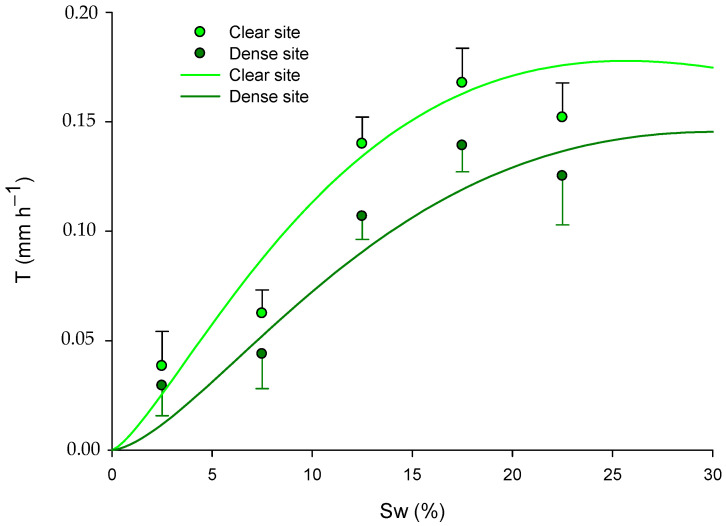
Fitted models for green leaves explaining the transpiration rate (T; mm h^−1^) in relation to soil water (Sw; %) and site (pine competition). The models are presented for the long-term period, with data across all seasons: for the dense site, $T={e^{\left(-5.82-0.053Sw\right)}\;}^{\;}{Sw}^{1.60}$; and for the clear site, $T={e^{\left(-4.78-0.053Sw\right)}}^{\;}{Sw}^{1.35}$. Mean values of transpiration rate ± standard error (mm h^−1^) are also presented in the graph at soil water content intervals of 2.5%, 7.5%, 12.5%, 17.5%, and 22.5%.

In accordance with the previous results in Section 2.3 and Section 3.2, the model’s response varied depending on the competition effects of the pines (site) in spring and summer, as evidenced by the significance of the dummy variable “S” (Table 5). Furthermore, after analyzing the yearly model using all the available data, we found that with a certain increase in Sw, the predicted transpiration was significantly higher in the clear site (Table 5 and Figure 7).

From a statistical point of view, the site variable includes the confounding effects of competition for water and light, among other environmental variables, in each stand. Consequently, the transpiration could be explained in terms of the analyzed parameters, mainly the PAR and the Sw.

Our results initially confirmed that the PAR increased transpiration in all the seasons, attributable to the marked heliophilia of esparto grass [17]. Thus, our findings align with the general rule that individuals growing in light exhibit higher transpiration compared to suppressed individuals in the shadows of neighbors [10]; this is a hypothesis previously confirmed by Gasque and García-Fayos [17] in their study of the effects of Aleppo pine on *M. tenacissima*.

However, since the open forests exhibit significantly higher soil humidity during the drought period despite their lower water retention capacity (Table 6), the site effects could also, in part, be attributed to competition for water. We can consider that both stands have similar precipitation and runoff levels (as they are mixed), and the effects of water interception by pine crowns could be negligible, because esparto grass does not grow completely under the treetops. Furthermore, the absence of differences observed in the measured PAR between the sites in spring, and the lack of a significant effect of the competition index on PAR, support the hypothesis that a portion of the confounding effect should be due to water competition.

As noted by Ramirez et al. [24], *M. tenacissima* leaves exposed to high irradiance exhibited a more intense transpiration response to light, but only under conditions of higher water availability. Although we did not measure transpiration in *Pinus halepensis*, studies by [16,46] have confirmed that Aleppo pine does not completely close its stomata during the summer and transpires at a rate of about 0.2 mm day^−1^; thus, it potentially competes for water.

Additionally, during the summer, the significantly lower PAR measured in the closed stands, attributed to shade from nearby pines, likely created a positive effect on esparto (“photo-protective effect”, [33]). This is a scenario that allows plants to cope with high irradiance and water stresses in semi-arid environments [23], similar to that resulting from the structural photo-protection of self-shading by its own leaves [24].

Thus, our findings also align with the postulate that the variation in seasonal transpiration depends, in part, on soil water availability [10]. Competition influences water availability, especially within species coexisting in semi-arid environments, affecting the transpiration of dominated individuals [10,17,29,47].

## 3. Materials and Methods

### 3.1. Study Area

**Figure 8:**
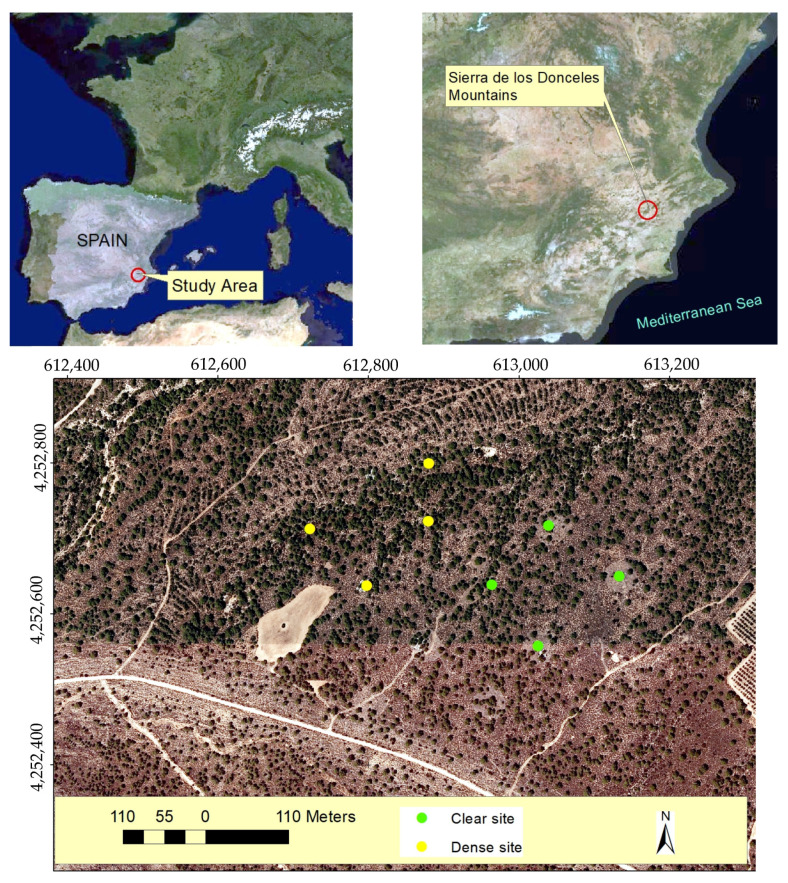
Location of the study area in the south of Spain (Sierra de los Donceles Mountains) and the sampled esparto grass where transpiration measurements were conducted in both clear and dense stands. The coordinates are in UTM (m), using the ETRS89 reference system.

The study was conducted in pine forests dominated by *P. halepensis* as the tree species in the canopy or overstory, with *M. tenacissima* L. as the main herbaceous species. These vegetal formations are situated in the Sierra de los Donceles Mountain (in the southeast of the Iberian Peninsula, Spain; Figure 8). Other species present in the understory form an evergreen sclerophyllous layer, including *Rhamnus lycioides* L., *Rosmarinus officinalis* L., *Pistacia lentiscus* L., and *Quercus coccifera* L. [48].

The climate is classified as Mediterranean semi-arid, type BSk, according to the Köppen–Geiger climate classification system. The area falls within the meso-Mediterranean bioclimatic type, with an average annual temperature and precipitation of 15.8 °C and 327.6 mm, respectively (1990–2022 period; meteorological data provided by the Spanish Meteorological Agency, AEMET, Madrid). The region experiences a large thermal amplitude, resulting in an average annual temperature ranging from 42.6 °C in August to −1.2 °C in January. Precipitation occurs erratically throughout the year, primarily in short periods marked by intense storm events.

The Donceles mountain range is characterized by dolomitic limestone formations dating back to the Jurassic period, with altitudes ranging from 504 to 808 m.a.s.l. The pine forest in the study area is positioned on a mid-slope area, facing north. Based on the edaphic properties, the soil was classified as Aridisol (Lithic Haplocalcids) according to the USDA Soil Taxonomy [49], owing to limited soil moisture available for plant growth and the accumulation of carbonates. The profile extends from a depth of 10 to 20 cm.

The density of Aleppo pine stands in this forest is highly heterogeneous (Table 6). Clear stands exhibit a low density of trees (88 ± 12 pines ha^−1^), with the herbaceous vegetation of esparto grass dominating the understory (with a cover of 65%). In contrast, the dense site represents a closed pine forest with a high density of Aleppo pine trees. Because of this, the competition index (CI) was significantly higher in the pine-dominated forests.

**Table 6:** Vegetation characteristics and edaphic parameters in soils defining the site quality of the two forest sites. Errors: standard error. N° of soil samples: 6 per site.

Characteristics	Closed Stands	Open Stands
Vegetation ^1^		
Tree density (pines ha^−1^)	308 ± 40	88 ± 12
Basal area (m^2^ ha^−1^)	5.0 ± 0.7	3.1 ± 1.2
Canopy cover (%)	42.3 ± 12.1	18.3 ± 21.1
Mean crown diameter	4.54 ± 2.46	5.86 ± 2.09
Volume growth (m^3^ ha^−1^ year^−1^)	0.4 ± 0.1	0.2 ± 0.1
Esparto cover (%)	32 ± 23	65 ± 14
CI (m m^−1^)	0.22 ± 0.01	<0.01 ± 0.001
Soils ^2^		
Soil pH	8.3 ± 0.1	8.4 ± 0.1
Sand (%)	46.3 ± 7.5	55.4 ± 4.6
Clay (%)	37.0 ± 7.9	31.4 ± 3.8
Organic matter (%)	4.2 ± 0.6	3.4 ± 0.7
Total N (%)	0.2 ± 0.01	0.1 ± 0.01
Available P (ppm)	5.0 ± 0.9	3.0 ± 1.2
Water storage capacity (mm)	54.4 ± 6.5	46.5 ± 4.4

^1^ Data from Pérez-Anta [50]. ^2^ Texture was determined using a Bouyoucos densimeter; pH using a pH Meter; organic matter (%) using the Walkley–Black method; total N (%) using the Kjeldahl method; and bioavailable P following the method of Olsen.

The soil analysis indicated the presence of highly alkaline soils (pH: 8–8.5) with a clay content exceeding 30% throughout the profile (Table 6). The water storage capacity (mm) analysis, calculated following the method of Domínguez et al. [51], which is based on soil texture, resulted in 54.4 ± 6.5 mm for the closed stands and 46.5 ± 4.4 mm for the open site (Table 6). Thus, the soils in the closed site are capable of retaining a higher amount of water due to their higher content of clays and organic matter. Retention capacity is also closely related to permeability, which serves as an indicator of the ease of root penetration. Additionally, the nutrient content (N, P) in the soils situated in the closed stands presented higher values than those in the open woodlands, indicating higher soil quality.

### 3.2. Experimental Layout and Sampling of Bushes

Diurnal patterns of water exchange in the green and senesced leaves were monitored from August 2020 to March 2021 on 14 sampling days, spanning the four seasons, thus reflecting the period of water stress (summer), the period of growth and return to dormancy (autumn), dormancy (winter), and the season of activate growth (spring) [35].

To carry this out, eight esparto bushes were chosen as samples for the leaf-level transpiration measurements. The bushes were growing under two levels of pine–esparto competition, encompassing both clear and dense stands, and thereby experiencing different water absorption effects from nearby pines. All the sampling bushes were less than 1 km apart, ensuring that they were growing under the same rainfall, as well as at a similar altitude and level of exposure (flat surfaces). Moreover, as can be seen in Figure 8, the dense and closed stands appear mixed in the pine woodland. Four of the measured esparto grass samples were selected from the clear site, while the remaining four were chosen from the dense site.

The esparto grasses in the dense site were located within a circular area with a 5 m radius from the pine competitor. In the open site, the sampled esparto bushes were isolated from their pine competitors (>10 m). The tussocks were selected while maintaining a distance greater than 2 m from the surrounding esparto grass to prevent intraspecific alterations in their water balance. To evaluate the level of pine competition, a competition index (CI; m m^−1^) was calculated as the quotient of the perimeter of each competitor tree, divided by the distance separating it from the bush (see Table 6).

We additionally assessed transpiration in two bush maturity groups based on their perimeter at the base (Pb), because younger esparto grass typically exhibits smaller cover size [22]: young (<70 cm perimeter of the base) and mature (>130 cm perimeter) esparto grass; young (Pb < 70 cm) and mature (Pb > 130 cm) esparto grass. Two of the esparto grasses in each site were young, and the other two were mature.

Within each selected tussock, tillers were randomly chosen on each sampling day and categorized as either green leaves (in their first or second growing season) or senescent leaves (displaying orange to yellow colors). The aim was to measure transpiration based on the type of leaf. No differences existed between the soils or slopes of the two studied sites.

### 3.3. Measurements of Transpiration at Leaf Level

**Figure 9:**
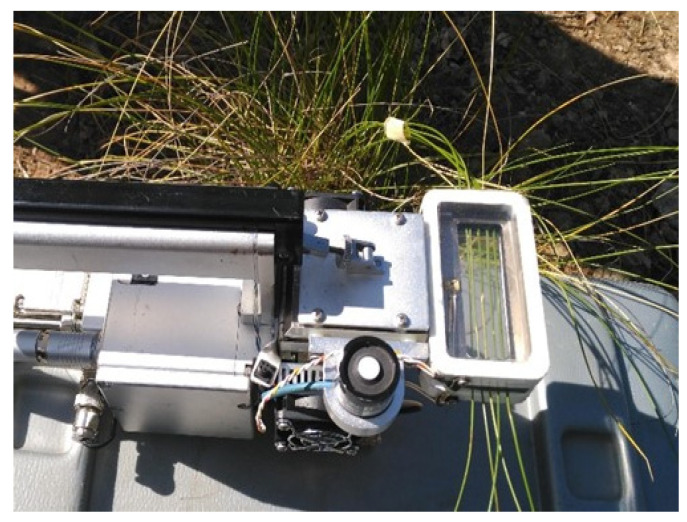
Measurement of transpiration in *M. tenacissima* L. leaves using the LI-6400XT portable equipment (LI-COR Inc., Lincoln, NE, USA). The setup involved the use of the 6400-07 Needle Chamber, which is specifically designed for needle-like leaves. The sample consisted of 5 segments of leaves.

Transpiration at the leaf level was quantified using LI-6400 XT equipment, which incorporates a camera for the measurement of water fluxes in needles (6400 07 Needle Chamber; LI-COR Inc., Lincoln, NE, USA). The measurements were conducted three times a day (at approximately 8:00, 12:00, and 16:00 UTC) to mitigate the influence of temperature fluctuations during daylight hours on transpiration. This approach was also chosen because transpiration in esparto grass is maximized around midday [22,50], which is a pattern that is characteristic of plants with C3 metabolism [52]. Each replica consisted of five leaves taped together; these replicas were placed in the IRGA chamber (Figure 9). Three repeated measures were taken for each hour, and the mean was used for subsequent statistical analysis. Thus, the total dataset included 3 times day^−1^ × 2 types of leaf × 8 bushes × 14 sampling days. Transpiration was registered in the leaves at full sunlight to avoid photo-inhibition effects. Water exchange was calculated based on the projected leaf area by employing the formula integrated into the LI-COR 6400/XT operating software:

$$T=\frac{F\;(W_{s}-W_{r})}{100\;S\;\left(100-W_{s}\right)}$$

where *T* represents transpiration (mol H_2_O m^−2^ s^−1^), *F* is the air flow entering the chamber (μmol air s^−1^), *W_s_* and *W_r_* are the mole fractions of water in the sample and the reference flows, respectively (mmol H_2_O (mol air)^−1^), and *S* denotes the measured leaf area (cm^2^). Afterward, water exchange was expressed as the transpiration rate in mm h^−1^, taking into account the weight of 1 mmol of H_2_O (0.018016 g) and 1 L of water = 1 mm m^−2^. Thus, transpiration represents the rate of water exchange per unit of foliar area (m^−2^). The leaf segments used in the measurements were harvested, and their projected area was determined using leaf area measurement equipment (WD-E3; Delta-T Devices, Cambridge, UK). Considering that esparto grass folds its leaves into a cylinder, the only viable approach was to assess the projected leaf area in the closed position [27].

### 3.4. Biometric Characterization of Sampled Bushes: Plant Structure

To determine the biometry of each sampled esparto grass plant, at the conclusion of the experimental campaign, the bushes used for sampling were extracted from the soil. Subsequently, in the laboratory, the esparto components—green leaves, senescent leaves, dead leaves, and roots—were separated. The dry biomass of each foliar component (green or senescent leaves) was obtained by drying the samples in an oven (65 °C for 48 h). The samples were weighed using a precision balance (sensitivity ± 0.01 g; Kern EW, Kern & Sohn GmbH, Balingen, Germany). Moreover, the dimensions of the root (root depth and root length) were determined using a scaler ruler (±cm). To determine the total projected leaf area of each bush, a subsample of each leaf type was selected to calculate the specific leaf area (SLA), resulting in an SLA of 10.8 ± 0.9 cm^2^ g^−1^ for the study area. Afterward, the total dry weight of each leaf type was multiplied by the SLA to obtain the total leaf area per leaf type for the bush [50].

### 3.5. Microclimatic Soil Measurements and Environmental Conditions

Soil moisture content (Sw; %) and soil temperature (Ts; °C) were measured at a depth of 5 cm using a moisture probe (Theta Probe ML2x, Delta-T Devices, Cambridge, UK) and a temperature probe (TMC20-HD, Onset Computers, Bourne, MA, USA), respectively. Data collection was conducted beneath each sampled esparto grass during the transpiration measurement period. The data were recorded in dataloggers (CR1000, Campbell Scientific, Logan, UT, USA). Additionally, the photosynthetically active radiation (PAR; µmol m^−2^ s^−1^) was recorded in situ using a levelable quantum sensor integrated into the LI6400 (LI-190, LI-COR Inc., Lincoln, NE, USA). The vapor pressure deficit (VPD; kPa) was calculated using parameters that were also measured inside the chamber connected to the LI6400 equipment to provide the difference between the vapor pressure at the saturation point of the plant and the vapor pressure of the air.

### 3.6. Statistical Analysis

First, a correlation matrix was conducted to detect significant relationships (at the 95% confidence level; *p* < 0.05) between T, soil microclimatic variables (Sw, Ts), and environmental parameters (PAR, VPD).

The influence of the considered factors on the transpiration rate (T; mm h^−1^) was examined using nested linear mixed models (LMMs). While the measurements were independent, with distinct leaves measured on each sampling day, it was necessary to consider repeated measurements of the same subject (bush) within a sample of leaves. The first model included as the fixed components the leaf type (2 levels: green or senescent), site (2 levels: clear or dense), season (4 levels based on sampling time: winter, spring, summer, and autumn), and bush maturity (2 levels: young and mature esparto grass), along with their interactions. This model aimed to detect differences between the green and senescent leaves in relation to the main factors.

Afterward, the model was readjusted by removing the type of leaf as a fixed effect; then, only the transpiration of the green leaves was considered as the dependent variable (to isolate and highlight the effects of the main factors on leaves with higher physiological activity, i.e., the green leaves).

The two models incorporated the sampled bush (8 levels) nested within the site and the heteroscedastic variance across the sampling dates as random effects. In the models, the sampling “bush” was nested within the “site” since different shrubs were measured in each woodland, and “bush” was considered a random factor since the selected bushes were a random sample from the total esparto grass (randomized design, [53]). Thus, this model represents a linear mixed-effects model with fixed and nested random effects. The effects of “between factors” on the response variable “within each season” were also analyzed, taking into account the seasonal data.

The random variance components were estimated using restricted maximum likelihood estimation (RMLE). The model selection was based on the Bayesian information criterion (BIC) and the Akaike information criterion (AIC). The mean values were compared using Fisher’s LSD post-hoc test (α = 0.05) if the factors were significant. The mixed models were fitted using the nlme package in R [54]. In addition, we explored the relationships between the rate of water exchange (T; mm h^−1^) and the soil water content (Sw; %). These models were conducted using the entire dataset for both types of leaves across all seasons, employing the following combined exponential power model (Model 1):

$$T={exp}^{\left(\beta_{0}+\beta_{0}^{\prime }S\right)+\left(\beta_{1}+\beta_{1}^{\prime }S\right)\times Sw}{Sw}^{\left(\beta_{2}+\beta_{2}^{\prime }S\right)}\times \varepsilon$$

This model, which is a generalization of the power function [55], captures the relationship that occurs when transpiration increases based on soil humidity but follows a hump-shaped pattern skewed to the right. In Model 1, the influence of the competence (site) on transpiration was incorporated through the use of a dummy variable “S” (S = 0 for the clear site, and S = 1 for the dense site). The models were estimated for both short-term (seasonal variations in transpiration) and long-term periods (annual data aggregated across all seasons). The selection of the significant parameters in Model 1 involved logarithmic transformation (linearization) and stepwise regression, which facilitated the comparison of two regression lines between the two sites across different seasons. The coefficients with a *p*-value < 0.05 were deemed significant. The regressions were evaluated based on the F ratio (*p* < 0.05) and the adjusted R^2^ [56]. The values with absolute DIFT > (2$\times \sqrt{\frac{n}{p}}$) were considered influential points (p is the number of coefficients, and n is the number of data points [57]) and were removed. The regressions were fitted using Statgraphics Centurion vers. XVIII^®^ software (Statgraphics Technologies Inc., The Plains, VA, USA). Using this software, we also analyzed the correlations between the measured variables and the transpiration rates.

## 4. Conclusions

While it has been challenging to isolate the specific importance or individual contribution of competition for light or water with regard to the effects of pines on esparto grass (as these factors constitute a confounding effect), our results not only confirmed the positive relationship between PAR and transpiration in esparto but also revealed the correlations between water exchange from leaves and soil availability. These correlations are influenced by competition from pines, seasonality, and the maturity of the bushes.

Our results suggest that in stands where esparto occupies the herbaceous layer under elevated competition with pines, an increase in drought due to climatic change could contribute to the loss of esparto cover in Mediterranean climates. This vulnerability is highlighted by the finding that young esparto grass is particularly susceptible to competition from pines. In this regard, to restore mixed pine–esparto grass ecosystems in the Mediterranean basin, reforestation programs should aim to reduce the percentage of pines in the reforested areas and to incorporate esparto. This approach helps to mitigate the excessive competition affecting esparto grass, which could jeopardize its survival, particularly in the context of climate change.

Moreover, further investigations of pine–esparto formations are necessary to deepen our understanding of the crucial ecophysiological processes related to water use efficiency and carbon sequestration in semi-arid Mediterranean woodland. In this sense, the intriguing issue involving the isolation of effects due to competition for light or water (and other factors) could be addressed in future studies. This would help to better explain the behavior of *M. tenacissima* in mixed stands, which grows in an understory dominated by pines, and to aid in the understanding of the future of this species in a scenario of climate change.

## Figures and Tables

**Table 1:** Spearman correlation coefficients between transpiration (T; mm h^−1^) of green leaves, competition index (CI; m m^−1^), soil water content (Sw; %), soil temperature (Ts; °C), photosynthetic active radiation (PAR; µmol m^−2^ s^−1^), and vapor pressure deficit (VPD; kPa). In green background: correlations significantly different from zero, with a confidence level of 95% (*p* < 0.05).

Seasons	Variables	CI (m m^−1^)	Sw (%)	Ts (°C)	PAR (µmol m^−2^ s^−1^)	VPD (kPa)	T (mm h^−1^)
Summer 2020	CI		−0.404	−0.339	−0.199	−0.020	−0.298
	Sw	−0.404		−0.396	0.194	−0.322	0.536
	Ts	−0.339	−0.396		0.454	0.432	−0.471
	PAR	−0.199	0.194	0.454		0.321	0.517
	VPD	−0.020	−0.322	0.432	0.321		0.234
	T	−0.298	0.536	−0.471	0.517	0.234	
Autumn 2020	CI		−0.093	−0.154	−0.032	−0.123	−0.088
	Sw	−0.093		−0.583	0.007	−0.034	0.572
	Ts	−0.154	−0.583		0.049	−0.035	−0.426
	PAR	−0.032	0.007	0.049		0.210	0.438
	VPD	−0.123	−0.034	−0.035	0.210		−0.165
	T	−0.088	0.572	−0.426	0.438	−0.165	
Winter 2020–2021	CI		−0.075	−0.058	0.025	−0.171	0.016
	Sw	−0.075		−0.407	0.134	0.035	−0.302
	Ts	−0.058	−0.407		−0.229	0.144	0.579
	PAR	0.025	0.134	−0.229		0.556	0.399
	VPD	−0.171	0.035	0.144	0.556		0.345
	T	0.016	−0.300	0.579	0.399	0.345	
Spring 2021	CI		−0.243	−0.135	−0.052	−0.201	−0.471
	Sw	−0.243		−0.423	0.187	0.154	0.360
	Ts	−0.135	−0.423		0.135	0.567	0.218
	PAR	−0.052	0.187	0.135		0.204	0.632
	VPD	−0.201	0.154	0.567	0.204		0.134
	T	−0.470	0.360	0.218	0.632	0.134	

**Table 2:** Summary of statistics for the linear mixed model (LMM) describing the influence of leaf type on the total transpiration rate (T, mm h^−1^) in the two sites across all seasons, including the effect of the maturity of the bush. Results are presented with the dependent variable as transpiration measured in both types of leaves (greens and senescent; entire dataset, n = 672). * Effects considered significant if *p* < 0.05 (95% probability).

	Seasons				
Main Effects	Spring	Summer	Autumn	Winter	Yearly
Leaf	<0.001 *	0.533	<0.000 *	0.001 *	<0.001 *
Leaf × Season	-	-	-	-	<0.001 *
Leaf × Site	0.858	0.687	0.571	0.873	0.317
Leaf × Maturity	0.322	0.223	0.447	0.784	0.295
Leaf × Season × Site	-	-	-	-	0.679
Leaf × Season × Maturity	-	-	-	-	0.724

**Table 3:** Summary of statistics for the linear mixed model (LMM) describing the influence of fixed factors of site and maturity on the total transpiration rate in green leaves (T, mm h^−1^; n = 336) across all seasons. * Effects are significant if *p* < 0.05 (95% probability).

	Seasons				
Main Effects	Spring	Summer	Autumn	Winter	Total Values
Season	-	-	-	-	<0.001 *
Site	0.026 *	0.003 *	0.625	0.702	0.088
Maturity	0.512	0.098	0.775	0.240	0.305
Season × Site	-	-	-	-	0.126
Season × Maturity	-	-	-	-	0.164
Site × Maturity	0.086	0.021 *	0.399	0.617	0.121
Season × Site × Maturity	-	-	-	-	0.607

**Table 4:** Significant parameters (±standard error), model significance (*p*), and goodness of fit (adjusted R^2^) in the exponential power model (Model 1), fitted for seasonal and annual periods. The model explains the transpiration rate (T; mm h^−1^) as a function of soil water content (Sw; %) and site (S): $T={exp}^{\left(\beta_{0}+\beta_{0}^{\prime }S\right)+\left(\beta_{1}+\beta_{1}^{\prime }S\right)\times Sw}{Sw}^{\left(\beta_{2}+\beta_{2}^{\prime }S\right)}$; n.s. = non-significant (*p* > 0.05; 95% probability). SEE: standard error of estimation; R^2^(%): coefficient of determination.

Seasons	Leaves	$\beta_{0}$	$\beta_{0}^{\prime }$	$\beta_{1}$	$\beta_{1}^{\prime }$	$\beta_{2}$	$\beta_{2}^{\prime }$	*p*	SEE	$R^{2}$(%)
Summer 2020	Greens	−8.62 ± 0.88	5.03 ± 1.05	n.s.	n.s	4.24 ± 0.81	−4.18 ± 0.91	0.00	1.04	34.9
	Senescent	−3.91 ± 0.08	n.s.	n.s.	n.s.	n.s.	n.s.	n.s.		
Autumn 2020	Greens	−5.64 ± 0.27	0.19 ± 0.09	n.s.	n.s.	1.26 ± 0.11	n.s.	0.00	0.57	48.5
	Senescent	−3.97 ± 0.10	n.s.	n.s.	n.s.	n.s.	n.s.	n.s.		
Winter 2020–2021	Green	−1.67 ± 0.23	n.s.	−0.048 ± 0.014	n.s.	n.s.	n.s.	0.00	0.39	23.8
	Senescent	−3.96 ± 0.14	n.s.	n.s.	n.s.	n.s.	n.s.	n.s.		
Spring 2021	Green	−2.14 ± 0.89	n.s.	n.s.	0.028 ± 0.010	n.s.	n.s.	0.00	0.31	23.6
	Senescent	−3.52 ± 0.09	n.s.	n.s.	n.s.	n.s.	n.s.	n.s.		
Yearly (all data)	Greens	−5.82 ± 0.29	1.04 ± 0.35	−0.053 ± 0.023	n.s.	1.60 ± 0.21	−0.25 ± 0.15	0.00	0.90	46.0
	Senescent	−3.92 ± 0.06	n.s.	n.s.	n.s.	n.s.	n.s.	n.s.		

**Table 5:** Fitted models account for each seasonal period, explaining the transpiration rate (T; mm h^−1^) in relation to soil water content (Sw; %), leaf type (green, senescent), and site (S): $T={exp}^{\left(\beta_{0}+\beta_{0}^{\prime }S\right)+\left(\beta_{1}+\beta_{1}^{\prime }S\right)\times Sw}{Sw}^{\left(\beta_{2}+\beta_{2}^{\prime }S\right)}$; n.s. = non-significant (*p* > 0.05; 95% probability). Average values of soil temperature (Ts; °C), soil water content (Sw; %), photosynthetic active radiation (PAR; µmol m^−2^ s^−1^), and vapor pressure deficit (VPD; kPa) within each adjustment period are denoted by different letters, indicating significant differences (Fisher’s LSD test, 95% probability, α = 0.05).

Seasons	Leaf Type–Site	T (mm h^−1^)	Sw (%)	Ts (°C)	PAR (µmol m^−2^ s^−1^)	VPD (kPa)
Summer 2020	Green–dense	$T={e^{\left(-8.62\right)}}^{\;}{Sw}^{4.24}$	3.4 ± 0.4	32.9 ± 0.5	959 ± 39	5.2 ± 2.9
	Green–clear	$T={e^{\left(-3.60\right)}}^{\;}{Sw}^{0.06}$				
	Senescent	T=0.0200				
Autumn 2020	Green–dense	$T=e^{\left(-5.64\right)}\;{Sw}^{1.25}$	13.0 ± 0.3	17.8 ± 0.3	576 ± 24	2.7 ± 1.5
	Green–clear	$T={e^{\left(-5.45\right)}}^{\;}{Sw}^{1.25}$				
	Senescent	T=0.0189				
Winter 2020–2021	Green–dense	$T={e^{\left(-1.67-0.048Sw\right)}\;}^{\;}$	16.3 ± 0.4	11.0 ± 0.5	458 ± 37	1.6 ± 0.6
	Green–clear	$T={e^{\left(-1.67-0.048Sw\right)}\;}^{\;}$				
	Senescent	T=0.0191				
Spring 2021	Green–dense	$T={e^{\left(-2.14\right)}\;}^{\;}$	11.9 ± 0.7	12.8 ± 0.8	198 ± 64	1.4 ± 0.3
	Green–clear	$T={e^{\left(-2.14+0.027Sw\right)}\;}^{\;}$				
	Senescent	T=0.0296				
Yearly	Green–dense	$T={e^{\left(-5.82-0.053Sw\right)}}^{\;}{Sw}^{1.60}$	11.8 ± 6.5	18.9 ± 9.5	596 ± 490	2.8 ± 2.1
(all data)	Green–clear	$T={e^{\left(-4.78-0.053Sw\right)}}^{\;}{Sw}^{1.35}$				
	Senescent	T=0.0198				

## Data Availability

Datasets are available on request to authors.

## References

[1] Pugnaire F.I., Armas C., Maestre F.T. (2011). Positive plant interactions in the Iberian Southeast: Mechanisms, environmental gradients, and ecosystem function. J. Arid. Environ..

[2] Wu W., Li H., Feng H., Si B., Chen G., Meng T., Li Y., Siddique K.H.M. (2021). Precipitation dominates the transpiration of both the economic forest (*Malus pumila*) and ecological forest (*Robinia pseudoacacia*) on the Loess Plateau after about 15 years of water depletion in deep soil. Agric. For. Meteorol..

[3] Sperlich D., Chang C.T., Peñuelas J., Gracia C., Sabaté S. (2015). Seasonal variability of foliar photosynthetic and morphological traits and drought impacts in a Mediterranean mixed forest. Tree Physiol..

[4] Lu L., Zhang D., Zhang J., Zhang J., Zhang S., Bai Y., Yang S. (2023). Ecosystem Evapotranspiration Partitioning and Its Spatial–Temporal Variation Based on Eddy Covariance Observation and Machine Learning Method. Remote Sens..

[5] Kozlowski T.T., Pallardy S.G. (1997). Transpiration and Plant Water Balance. Physiology of Woody Plants.

[6] Buckley T.N. (2017). Modeling Stomatal Conductance. Plant Physiol..

[7] Landsberg J.J., Gower S.T., Landsberg J.J., Gower S.T. (1997). 4—Forest Hydrology and Tree–Water Relations. Applications of Physiological Ecology to Forest Management.

[8] Zhu Y., Cheng Z., Feng K., Chen Z., Cao C., Huang J., Ye H., Gao Y. (2022). Influencing factors for transpiration rate: A numerical simulation of an individual leaf system. Therm. Sci. Eng. Prog..

[9] Li X., Zhai J., Sun M., Liu K., Zhao Y., Cao Y., Wang Y. (2024). Characteristics of Changes in Sap Flow-Based Transpiration of Poplars, Locust Trees, and Willows and Their Response to Environmental Impact Factors. Forests.

[10] Smith W.K., Hinckley T.M. (1995). Resource Physiology of Conifers: Acquisition, Allocation and Utilization.

[11] Pallardy S.G., Čermák J., Ewers F.M., Kaufmann M.R., Parker W.C., Sperry J.S., Smith W.K., Hinckley T.M. (1995). Water transport dynamics in trees and stands. Resource Physiology of Conifers.

[12] Buckley T.N. (2019). How do stomata respond to water status?. New Phytol..

[13] Vitale M., Anselmi S., Salvatori E., Manes F. (2007). New approaches to study the relationship between stomatal conductance and environmental factors under Mediterranean climatic conditions. Atmos. Environ..

[14] Han T., Feng Q., Yu T., Liu W., Ma J., Zhao C., Yang L., Zhang J., Li H. (2023). Contrasting response of water use efficiency to soil moisture availability: From leaf to ecosystem in an arid oasis. Ecol. Indic..

[15] Huang L., Zhang Z. (2016). Effect of rainfall pulses on plant growth and transpiration of two xerophytic shrubs in a revegetated desert area: Tengger Desert, China. Catena.

[16] Ungar E.D., Rotenberg E., Raz-Yaseef N., Cohen S., Yakir D., Schiller G. (2013). Transpiration and annual water balance of Aleppo pine in a semiarid region: Implications for forest management. For. Ecol. Manag..

[17] Gasque M.a., García-Fayos P. (2004). Interaction between *Stipa tenacissima* and *Pinus halepensis*: Consequences for reforestation and the dynamics of grass steppes in semi-arid Mediterranean areas. For. Ecol. Manag..

[18] Sanchez G., Puigdefabregas J. (1994). Interactions of plant growth and sediment movement on slopes in a semi-arid environment. Geomorphology.

[19] Belkhir S., Koubaa A., Khadhri A., Ksontini M., Smiti S. (2012). Variations in the morphological characteristics of *Stipa tenacissima* fiber: The case of Tunisia. Ind. Crops Prod..

[20] Redmann R.E. (1985). Adaptation of Grasses to Water Stress-Leaf Rolling and Stomate Distribution. Ann. Mo. Bot. Gard..

[21] Rivero R.M., Kojima M., Gepstein A., Sakakibara H., Mittler R., Gepstein S., Blumwald E. (2007). Delayed leaf senescence induces extreme drought tolerance in a flowering plant. Proc. Natl. Acad. Sci. USA.

[22] Ramírez D.A., Valladares F., Blasco A., Bellot J. (2006). Assessing transpiration in the tussock grass *Stipa tenacissima* L.: The crucial role of the interplay between morphology and physiology. Acta Oecol..

[23] Valladares F., Pugnaire F.I. (1999). Tradeoffs Between Irradiance Capture and Avoidance in Semi-arid Environments Assessed with a Crown Architecture Model. Ann. Bot..

[24] Ramírez D.A., Valladares F., Domingo F., Bellot J. (2008). Seasonal water-use efficiency and chlorophyll fluorescence response in alpha grass (*Stipa tenacissima* L.) is affected by tussock size. Photosynthetica.

[25] Cerdà A. (1997). The effect of patchy distribution of *Stipa tenacissima* L. on runoff and erosion. J. Arid. Environ..

[26] Lv X., Zhou G., Wang Y., Song X. (2016). Sensitive Indicators of Zonal Stipa Species to Changing Temperature and Precipitation in Inner Mongolia Grassland, China. Front. Plant Sci..

[27] Haase P., Pugnaire F.I., Clark S.C., Incoll L.D. (1999). Environmental control of canopy dynamics and photosynthetic rate in the evergreen tussock grass *Stipa tenacissima*. Plant Ecol..

[28] Krichen K., Vilagrosa A., Chaieb M. (2019). Divergence of functional traits at early stages of development in *Stipa tenacissima* populations distributed along an environmental gradient of the Mediterranean. Plant Ecol..

[29] Pugnaire F.I., Haase P. (1996). Comparative Physiology and Growth of Two Perennial Tussock Grass Species in a Semi-Arid Environment. Ann. Bot..

[30] Ghiloufi W., Quero J.L., García-Gómez M., Chaieb M. (2016). Potential impacts of aridity on structural and functional status of a southern Mediterranean *Stipa tenacissima* steppe. S. Afr. J. Bot..

[31] Han L., Liu L., Peng L., Wang N., Zhou P. (2023). Mixing of tree species with the same water use strategy might lead to deep soil water deficit. For. Ecol. Manag..

[32] Moreno-Gutiérrez C., Battipaglia G., Cherubini P., Saurer M., Nicolás E., Contreras S., Querejeta J.I. (2012). Stand structure modulates the long-term vulnerability of *Pinus halepensis* to climatic drought in a semiarid Mediterranean ecosystem. Plant Cell Environ..

[33] Ramírez D.A., Valladares F., Blasco A., Bellot J. (2008). Effects of tussock size and soil water content on whole plant gas exchange in *Stipa tenacissima* L.: Extrapolating from the leaf versus modelling crown architecture. Environ. Exp. Bot..

[34] Maestre F.T., Cortina J. (2005). Remnant shrubs in Mediterranean semi-arid steppes: Effects of shrub size, abiotic factors and species identity on understorey richness and occurrence. Acta Oecol..

[35] Balaguer L., Pugnaire F.I., Martínez-Ferri E., Armas C., Valladares F., Manrique E. (2002). Ecophysiological significance of chlorophyll loss and reduced photochemical efficiency under extreme aridity in *Stipa tenacissima* L.. Plant Soil.

[36] Haworth M., Marino G., Cosentino S.L., Brunetti C., De Carlo A., Avola G., Riggi E., Loreto F., Centritto M. (2018). Increased free abscisic acid during drought enhances stomatal sensitivity and modifies stomatal behaviour in fast growing giant reed (*Arundo donax* L.). Environ. Exp. Bot..

[37] Carignato A., Vázquez-Piqué J., Tapias R., Ruiz F., Fernández M. (2019). Variability and Plasticity in Cuticular Transpiration and Leaf Permeability Allow Differentiation of Eucalyptus Clones at an Early Age. Forests.

[38] Yang Y., Zhou Z., Li Y., Lv Y., Yang D., Yang S., Wu J., Li X., Gu Z., Sun X. (2020). Uncovering the role of a positive selection site of wax ester synthase/diacylglycerol acyltransferase in two closely related *Stipa* species in wax ester synthesis under drought stress. J. Exp. Bot..

[39] Li X., Huang Q., Mi X., Bai Y., Zhang M., Li X. (2018). Grazing every month minimizes size but boosts photosynthesis in *Stipa grandis* in the steppe of Inner Mongolia, China. J. Arid Land.

[40] Zhou A., Liu E., Liu J., Feng S., Gong S., Wang J. (2018). Characterization of increased cuticular wax mutant and analysis of genes involved in wax biosynthesis in *Dianthus spiculifolius*. Hortic. Res..

[41] Schuster A.C., Burghardt M., Riederer M. (2017). The ecophysiology of leaf cuticular transpiration: Are cuticular water permeabilities adapted to ecological conditions?. J. Exp. Bot..

[42] Ramírez D.A., Bellot J., Domingo F., Blasco A. (2006). Can water responses in *Stipa tenacissima* L. during the summer season be promoted by non-rainfall water gains in soil?. Plant Soil.

[43] Domingo F., Villagarcía L., Brenner A.J., Puigdefábregas J. (1999). Evapotranspiration model for semi-arid shrub-lands tested against data from SE Spain. Agric. For. Meteorol..

[44] Pugnaire F.I., Haase P., Incoll L.D., Clark S.C. (1996). Response of the Tussock Grass *Stipa tenacissima* to Watering in a Semi-Arid Environment. Funct. Ecol..

[45] El-Abbassi F.E., Assarar M., Ayad R., Bourmaud A., Baley C. (2020). A review on alfa fibre (*Stipa tenacissima* L.): From the plant architecture to the reinforcement of polymer composites. Compos. Part A Appl. Sci. Manuf..

[46] Raz-Yaseef N., Yakir D., Schiller G., Cohen S. (2012). Dynamics of evapotranspiration partitioning in a semi-arid forest as affected by temporal rainfall patterns. Agric. For. Meteorol..

[47] Kobayashi Y., Tanaka T. (2001). Water flow and hydraulic characteristics of Japanese red pine and oak trees. Hydrol. Process..

[48] García-Morote F., Martínez-García E., Andrés-Abellán M., Caballero E., Miettinen H., López-Serrano F. (2017). Direct Seeding of Pinus halepensis Mill. for Recovery of Burned Semi-Arid Forests: Implications for Post-Fire Management for Improving Natural Regeneration. Forests.

[49] USDA—United States Department of Agriculture (1999). Soil Taxonomy: A Basic System of Soil Classification for Making and Interpreting Soil Surveys.

[50] Pérez-Anta I. (2021). Efecto de *Stipa tenacissima* L. Sobre los Flujos de Agua del Sistema Suelo-Esparto en un Ecosistema Semiárido de Pinus Halepensis Mill. en el SE Peninsular: Implicaciones Para la Gestión Forestal. Ph.D. Thesis.

[51] Domingo Santos J.M., Fernández de Villarán San Juan R., Corral Pazos de Provens E., Rapp Arrarás I. (2006). Estimation of water retention capacity in soil: Corrections to the CRA pedotransfer formula. For. Syst..

[52] Leuning R. (1995). A critical appraisal of a combined stomatal-photosynthesis model for C3 plants. Plant Cell Environ..

[53] Hoshmand H.R. (2006). Design of Experiments for Agriculture and the Natural Sciences.

[54] Pinheiro J., Bates D., DebRoy S.S., Sarkar D., R Core Team (2022). Nlme: Linear and Nonlinear Mixed Effects Models. R Package Version 3.1-155. https://CRAN.R-project.org/package=nlme.

[55] Daniel C., Wood F.S. (1980). Fitting Equations to Data: Computer Analysis of Multifactor Data.

[56] Neter J., Kutner M., Wasserman W., Nachtsheim C. (1996). Applied Linear Statistical Models.

[57] Belsley D.A., Kuh E., Welsch R.E. (2013). Regression Diagnostics: Identifying Influential Data and Sources of Collinearity.

